# Hepatitis C Virus Sensitizes Host Cells to TRAIL-Induced Apoptosis by Up-Regulating DR4 and DR5 via a MEK1-Dependent Pathway

**DOI:** 10.1371/journal.pone.0037700

**Published:** 2012-05-25

**Authors:** Zhongfan Deng, Huijuan Yan, Jiajie Hu, Shengwei Zhang, Peng Peng, Qingzhen Liu, Deyin Guo

**Affiliations:** State Key Laboratory of Virology and The Modern Virology Research Centre, College of Life Sciences, Wuhan University, Wuhan, People's Republic of China; Utah State University, United States of America

## Abstract

**Background:**

Hepatitis C virus (HCV) is the leading cause of liver fibrosis, cirrhosis and hepatocellular carcinoma. It is believed that continuous liver cell apoptosis contributes to HCV pathogenesis. Recent studies have shown that HCV infection can sensitize host cells to TNF-related apoptosis-inducing ligand (TRAIL) induced apoptosis, but the mechanism by which HCV regulates the TRAIL pathway remains unclear.

**Methods and Results:**

Using a sub-genomic replicon and full length virus, JFH-1, we demonstrate that HCV can sensitize host cells to TRAIL-induced apoptosis by up-regulating two TRAIL receptors, death receptor 4 (DR4) and death receptor 5 (DR5). Furthermore, the HCV replicon enhanced transcription of DR5 via Sp1, and the HCV-mediated up-regulation of DR4 and DR5 required MEK1 activity. HCV infection also stimulated the activity of MEK1, and the inhibition of MEK1 activity or the knockdown of MEK1 increased the replication of HCV.

**Conclusions:**

Our studies demonstrate that HCV replication sensitizes host cells to TRAIL-induced apoptosis by up-regulating DR4 and DR5 via a MEK1 dependent pathway. These findings may help to further understand the pathogenesis of HCV infection and provide a therapeutic target.

## Introduction

TNF-related apoptosis-inducing ligand (TRAIL), also known as Apo2L, is a member of the tumor necrosis factor (TNF) super family [1]. However, in contrast to TNF-α and FasL, TRAIL typically induces apoptosis in transformed cells but not in healthy cells [2]. Furthermore, recent studies have shown that TRAIL also induces apoptosis in virus-infected cells, including cells infected with hepatitis B virus, hepatitis C virus (HCV), human immunodeficiency virus, and respiratory syncytial virus [3], [4], [5], [6], [7]. Thus, TRAIL may function as an immune surveillance factor by selectively killing transformed and virus-infected cells. There are four specific TRAIL receptors on the cell membrane, death receptor 4 (DR4; also known as TRAIL-R1 or TNFRSF10A), death receptor 5 (DR5; also known as TRAIL-R2, KILLER, or TNFRSF10B), decoy receptor 1 (DcR1; also known as TRAIL-R3, TRID, or TNFRSF10C) and decoy receptor 2 (DcR2; also known as TRAIL-R4 or TNFRSF10D). DcR1 and DcR2 are two decoy receptors that contain a TRAIL-binding domain but lack a functional death domain. TRAIL can also bind to osteoprotegerin, which is a soluble TNF receptor family member; however, it has low binding affinity at physiological conditions [8]. The binding of TRAIL to DR4 or DR5 results in receptor trimerization and cell apoptosis via the recruitment of Fas-associated death domain (FADD) to the C terminus of the receptors. FADD then recruits an apoptosis-initiator caspase (caspase 8 or caspase 10) via its death effecter domain to form the death-inducing signaling complex, which allows for auto activation of caspases [9]. The downstream signaling of activated caspase 8 or 10 is dependent on the cell type. In type 1 cells, caspase 3 is activated and cleaves many cellular proteins to induce apoptosis. In type 2 cells, the apoptosis signal is augmented by the mitochondrial pathway, which involves the activation of caspase 9 following the loss of the mitochondrial membrane potential and Apaf-1 activation [10].

HCV belongs to the *Flaviviridae* family. Its genome is an enveloped positive RNA of 9.6 kb in length, containing one large open reading frame (ORF). The large ORF is translated into one polyprotein which is cleaved into ten mature proteins including core, E1, E2, p7, NS2, NS3, NS4A, NS4B, NS5A, and NS5B protein. The core protein has a frame shift variant called the F protein [11]. Up to 3% of the global population is HCV positive, and approximately 80% of infected patients develop a chronic infection [12]. HCV-infected patients are typically treated with pegylated IFN-α plus ribavirin. However, half of the individuals infected with genetype 1 do not achieve sustained viral clearance [13]. Liver cell apoptosis has been observed in HCV-infected patients, and accumulating evidence suggests that liver cell apoptosis is involved in the pathogenesis of HCV infection [14]. It is believed that liver damage, at least in part, causes fibrosis of the liver [15]. Although a direct cytopathic effect of a high HCV viral load has also been reported [16], the current prevailing view is that the apoptosis of liver cells in chronically infected HCV patients is initiated by the host innate and adaptive immune response. Many studies examining HCV and apoptosis have been reported. Several of these studies focused on a single HCV protein, and other studies used a cell culture replicon. However, the results from these studies have been conflicting, and there is currently no consensus regarding the role of HCV in liver cell apoptosis [17].

Previous studies have reported that TRAIL and its functional receptors: DR4 and DR5 are up-regulated in the liver of HCV-infected patients [18], [19] and that the expression levels of DR4 and DR5 are elevated in some cases of HCV-related hepatocellular carcinoma (HCC) [20]. However, it has also been reported that the expression of DR4 or DR5 is not altered in the liver during HCV-mediated cirrhosis [21]. In cell culture system, it has been shown that the HCV core protein can enhance TRAIL-induced apoptosis in Huh7 cells, a hepatoma cell line normally insensitive to TRAIL [22]. Furthermore, it has also been recently shown that infection with the full-length virus JFH-1 can sensitize Huh7.5 cells to TRAIL-induced apoptosis without changing the expression level of the TRAIL receptors [4]. However, another study demonstrates that JFH-1 infection can trigger the expression of TRAIL and its functional receptors in a cell culture system [23]. Taken together, it still remains controversial if TRAIL receptors are elevated in HCV-infected cells, and the molecular mechanism that underlies the up-regulation of TRAIL receptors has not been examined to date.

A stable cell line, 9–13, that contains an HCV 1b sub-genomic replicon that lacks the genes for all structural proteins and the NS2 protein has been derived from Huh7 cells [24]. The first infectious HCV cell culture model, JFH-1, was developed by Dr. Wakita in 2005 [25]. These two HCV cell culture models have greatly aided HCV research. In the current study, we found that 9–13 cells were more sensitive to TRAIL-induced apoptosis than Huh7 cells and that the expression of DR4 and DR5 was significantly higher in 9–13 cells at both the mRNA and protein levels when compared with Huh7 cells. Elimination of the HCV replicon in the 9–13 cells decreased the expression of DR4 and DR5. Similarly, the expression level of DR4 and DR5 was elevated in Huh 7.5.1 cells when infected with JFH-1. Furthermore, HCV increased the transcription of DR5 via the transcription factor Sp1. HCV replication also stimulated the activity of MEK1 and MEK1 activity was required for DR4 and DR5 up-regulation in both 9–13 cells and JFH-1 infected Huh7.5.1 cells. Importantly, inhibition the activity or expression of MEK1 increased the replication of HCV. Together, these results demonstrate that HCV replication up-regulates DR4 and DR5 via a MEK1-dependent pathway, which results in the sensitization of host cells to TRAIL-induced apoptosis. These results suggest that MEK1 may be a negative regulator of HCV replication.

## Results

### HCV replicon-containing cells are more sensitive to TRAIL-induced apoptosis than naive cells

**Figure 1:**
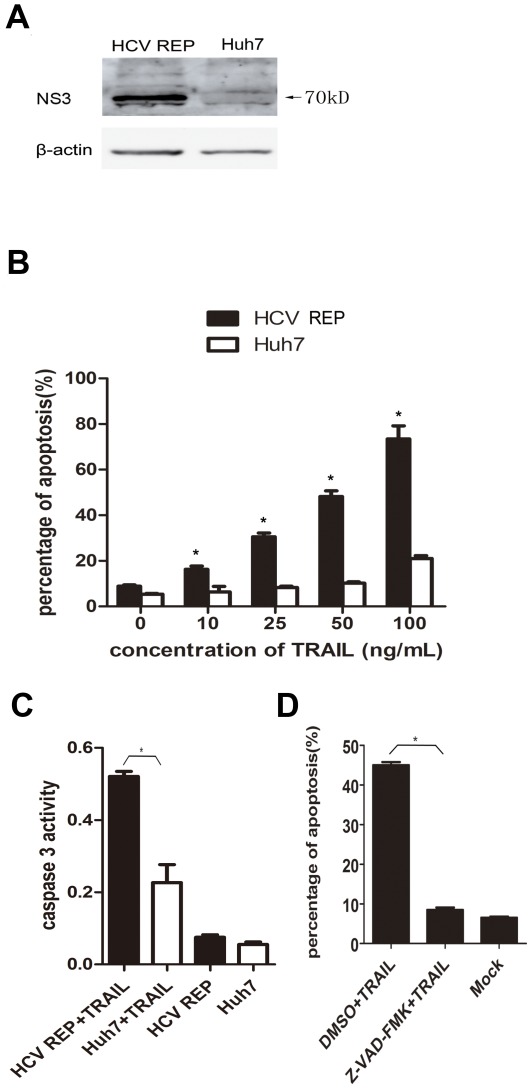
HCV replicon sensitizes host cells to TRAIL-induced and caspase-dependent apoptosis. (A) Cell lysates from HCV replicon-containing cells (9–13) and Huh7 cells were subjected to western blot analyses using a rabbit polyclonal antibody against HCV NS3/4A. (B) Huh7 and 9–13 cells were treated with TRAIL at different concentrations (10 ng/mL, 20 ng/mL, 50 ng/mL or 100 ng/mL) for 2 hr and stained with annexin V and PI. The proportion of apoptotic cells was measured using flow cytometry. (C) Huh7 and 9–13 cells were treated with 50 ng/mL TRAIL for 2 hr, and caspase 3 activity was measured using a Caspase 3 Activity Assay Kit. (D) 9–13 cells were treated with 20 µM Z-VAD-FMK (a pan-caspase inhibitor) or DMSO for 1 hr and subsequently treated with 50 ng/mL TRAIL for 2 hr. The mock-treated samples were untreated 9–13 cells. The proportion of apoptotic cells was measured using flow cytometry after the cells were stained with annexin V and PI. The data are presented with the SD from three independent experiments, and statistical significance was calculated by *t* test or two-way ANOVA, * indicates a *p* value less than 0.05.

TRAIL has the ability to induce apoptosis in some transformed cells [2]. However, Huh7 cells, a HCC cell line, are resistant to TRAIL-induced apoptosis [22]. Therefore, we tested if 9–13 cells (designated “HCV REP” in all figures), which were derived from the Huh7 cell line and contain a HCV 1b sub genomic replicon (Fig. 1A), were sensitive to TRAIL. When exposed to 25–100 ng/mL TRAIL for 2 hr, a large proportion of 9–13 cells underwent apoptosis, whereas only a small fraction of the TRAIL-treated Huh7 cells underwent apoptosis (Fig. 1B). Furthermore, the activity of caspase 3 was much greater in 9–13 cells than in Huh7 cells (Fig. 1C), indicating that the HCV replicon sensitized the host cells to TRAIL-induced apoptosis. The cell death induced by TRAIL was dependent on caspase activity because pre-treating 9–13 cells with the pan-caspase inhibitor, Z-VAD-FMK, completely abolished TRAIL-induced apoptosis (Fig. 1D).

### DR4 and DR5 are both up-regulated by HCV replication

**Figure 2:**
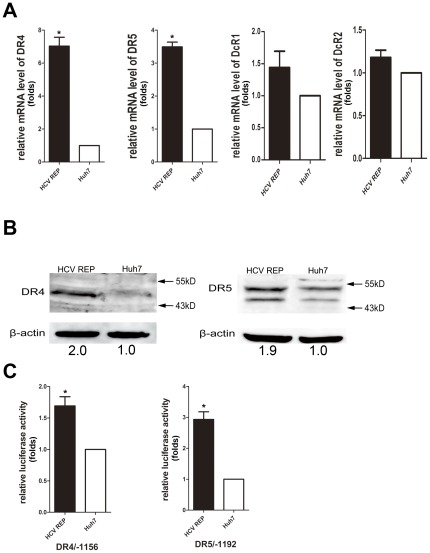
HCV replication up-regulates DR4 and DR5. (A) The mRNA level of DR4, DR5, DcR1, and DcR2 in 9–13 and Huh7 cells was measured using real-time RT-PCR. (B) Huh7 and 9–13 cell lysates were subjected to western blot analyses using a rabbit polyclonal antibody against DR4 or DR5. (C) The DR4 reporter plasmid (DR4/−1156; 100 ng) or DR5 reporter plasmid (DR5/−1192; 100 ng) was co-transfected with the *Renilla* luciferase reporter plasmid (100 ng) into 9–13 or Huh7 cells cultured in a 24-well plate. After 2 days, the cells were harvested, and the luciferase activity was measured. (A and C) The data from the 9–13 cells were normalized to Huh7 cells to directly show the fold induction caused by HCV. The data are presented with the SD from three independent experiments, and statistical significance was calculated by *t* test, * indicates a *p* value less than 0.05.

**Figure 3:**
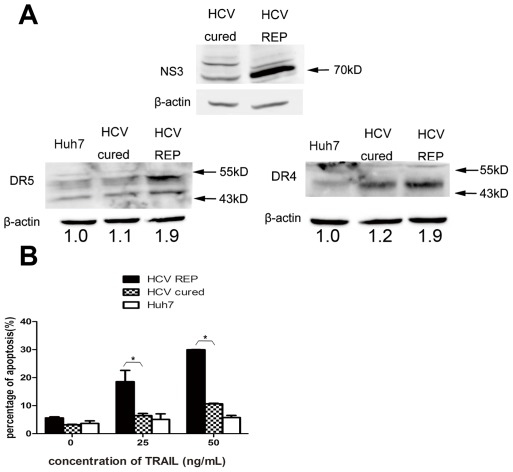
Up-regulation of DR4 and DR5 is HCV replication-dependent. (A) Lysates from Huh7, 9–13 and HCV-cured cells were subjected to western blot analysis using rabbit polyclonal antibodies against HCV NS3/4A, DR4 or DR5. (B) Huh7, 9–13 and HCV-cured cells were treated with 25 and 50 ng/mL TRAIL for 2 hr, and the proportion of apoptotic cells was measured using flow cytometry after the cells were stained with annexin V and PI. The data are presented with the SD from three independent experiments, and statistical significance was calculated by two-way ANOVA, * indicates a *p* value less than 0.05.

**Figure 4:**
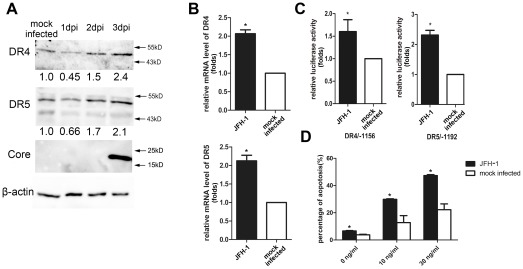
JFH-1 infection up-regulates the expression of DR4 and DR5. (A) Western blot analysis was performed to measure the expression of DR4 and DR5 in Huh7.5.1 cells infected with JFH-1 1, 2, 3 days post-infection (MOI 0.02). (B) Real-time PCR was performed to measure the mRNA levels of DR4 and DR5 in Huh7.5.1 cells infected with JFH-1 (MOI 0.02) 3 days post-infection. (C) The DR4 reporter plasmid (DR4/−1156; 100 ng) or DR5 reporter plasmid (DR5/−1192; 100 ng) was co-transfected with the *Renilla* luciferase reporter plasmid (50 ng) into Huh7.5.1 cells, 6 hr later, cells were infected with JFH-1 (MOI 0.5). After 3 days, the cells were harvested, and the luciferase activity was measured. The data from the infected cells were normalized to Huh7.5.1 cells to directly show the fold induction caused by HCV. (D) Huh7.5.1 cells were infected with JFH-1 (MOI 0.5), 3 days later, cells were treated with indicated concentration of TRAIL for 2 hr, and stained with annexin V and PI. The proportion of apoptotic cells was measured using flow cytometry. The data are presented with the SD from three independent experiments, and statistical significance was calculated by *t* test or two-way ANOVA, * indicates a *p* value less than 0.05.

We evaluated the mRNA levels of the TRAIL receptors using real-time PCR and found that the mRNA levels of DR4 and DR5 were higher in 9–13 cells when compared with Huh7 cells. Moreover, there was no significant change in the mRNA levels of DcR1 and DcR2 (Fig. 2A). Western blot analyses confirmed that DR4 and DR5 were up-regulated in 9–13 cells (Fig. 2B). The activity of the DR4 and DR5 promoters was then tested in both cell lines. The DR4 and DR5 promoter activities were significantly higher in 9–13 cells than in Huh7 cells (Fig. 2C). To verify that the up-regulation of DR4 and DR5 was due to HCV replicon replication, we eliminated the HCV replicon from 9–13 cells and analyzed the expression of DR4 and DR5. We treated 9–13 cells with 100 IU/mL IFN-α and cultured the cells for four passages. As shown in Fig. 3A, the replication of HCV was completely inhibited after the IFN-α treatment, and HCV RNA was barely detected by using real-time PCR (data not shown). These IFN-α treated cells were designated HCV-cured cells. The expression of DR4 and DR5 was significantly decreased in HCV-cured cells when compared with 9–13 cells (Fig. 3A). We analyzed the sensitivity of the HCV-cured cells to TRAIL-induced apoptosis and found that the HCV-cured cells were less sensitive to TRAIL when compared with 9–13 cells (Fig. 3B). These results demonstrate that HCV replication up-regulates DR4 and DR5 and sensitizes host cells to TRAIL-induced apoptosis. To further confirm the elevation of DR4 and DR5 levels is resulted from HCV infection, we infected Huh7.5.1 cells with JFH-1 and measured the expression of DR4 and DR5 1, 2, 3 days post-infection by western blotting. As shown in Fig. 4A, the expression of DR4 and DR5 was significantly up-regulated at the 3^rd^ day post-infection. Similar up-regulation was observed at mRNA level at the 3^rd^ day post-infection (Fig. 4B). The expression of DR4 and DR5 had a modest decrease at the 1^st^ day post-infection (Fig. 4A), this result suggests that HCV may have some method to inhibit the expression of DR4 and DR5, in order to prevent host cell apoptosis at the early period of infection. However, more experiments are needed to confirm this observation in future study. In addition, the promoter activity of DR4 and DR5 were significantly higher in the JFH-1 infected cells (Fig. 4C). And correlated with the up-regulation of TRAIL receptors, the infected cells were more sensitive to TRAIL-induced apoptosis (Fig. 4D).

### HCV-mediated up-regulation of DR4 transcription depends on the −464 to −384 bp region of the DR4 promoter

**Figure 5:**
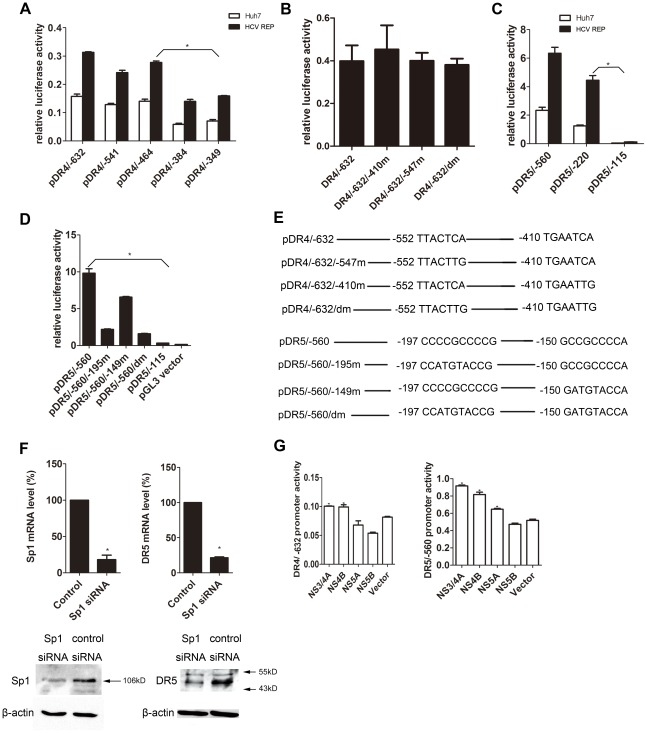
Transcriptional analysis of DR4 and DR5 in 9–13 cells. (A and C) Luciferase reporter plasmids (100 ng) containing different regions of the (A) DR4 or (C) DR5 promoter and the *Renilla* luciferase reporter plasmid (100 ng) were co-transfected into 9–13 or Huh7 cells. 2 days post-transfection, the cells were harvested, and luciferase activity was measured. (B and D) The indicated reporter plasmids (100 ng) illustrated in (E) were co-transfected with the *Renilla* luciferase reporter plasmid (100 ng) into 9–13 cells. 2 days post-transfection, the cells were harvested, and the luciferase activity was measured. (E) The mutations introduced into the AP-1 binding sites in pDR4/−632 and Sp1-binding sites in pDR5/−560 are illustrated. (F) The Sp1-specific siRNA or the control siRNA (100 pmol) was transfected into 9–13 cells cultured in 6-well plates. 2 days post-transfection, the Sp1 and DR5 RNA and protein levels were measured using real-time PCR and western blot analyses, respectively. (G) A plasmid expressing HCV NS3/4A, NS4B, NS5A or NS5B (600 ng) was individually co-transfected with either DR4/−632 or DR5/−560 (100 ng) and the *Renilla* luciferase reporter plasmid (100 ng) into Huh7 cells. 2 days post-transfection, the cells were harvested, and luciferase activity was measured. The data are presented with the SD from three independent experiments, and statistical significance was calculated by *t* test or two-way ANOVA, * indicates a *p* value less than 0.05.

We further studied the mechanism underlying the regulation of DR4 expression by HCV. The following reporter plasmids containing different regions of the DR4 promoter were constructed: pDR4/−632, pDR4/−541, pDR4/−464, pDR4/−384, and pDR4/−349. The promoter activity of these plasmids was compared in 9–13 and Huh7 cells. As shown in Fig. 5A, the 80 bp fragment between −464 and −384 bp of the DR4 promoter region was critical for DR4 transcription. A previous study has shown that −410/−404 is an AP-1 binding site that regulates the transcription of DR4 [26]. However, a point mutation in this AP-1 binding site (Fig. 5E) failed to influence the promoter activity of DR4 in 9–13 cells (Fig. 5B).

### HCV replicon enhances transcription of DR5 via Sp1

To elucidate the mechanism underlying HCV-mediated regulation of DR5, we constructed several reporter plasmids containing different regions of the DR5 promoter (pDR5/−560, pDR5/−220, and pDR5/−115). The promoter activity of these plasmids was compared in 9–13 and Huh7 cells. As shown in Fig. 5C, the −220 bp fragment of the DR5 promoter was sufficient for promoter activity. These results suggest that the increased DR5 expression caused by the HCV sub-genomic replicon is dependent on the DR5 promoter and that the −220 bp fragment of the promoter is the minimal promoter. The −195/−189 and −149/−143 sites of the promoter have been shown to be two Sp1 binding sites, which are important for the transcription of DR5 [27]. A luciferase assay was then performed with the following mutant constructs: pDR5/−560/−195m, pDR5/−560/−149m, and pDR5/−560/dm (pDR5/−560/−195m,−149m) (Fig. 5E). Mutation of the each of the Sp1 binding sites decreased the activity of the promoter, and the pDR5/−560/dm had no promoter activity (Fig. 5C). To further confirm that Sp1 is required for transcription of DR5 in 9–13 cells, we transfected 9–13 cells with a siRNA targeting Sp1. The expression of DR5 was detected using real-time PCR and western blot analyses 2 days post-transfection. The RNA and protein levels of DR5 were decreased when compared with cells transfected with control siRNA (Fig. 5F). These results suggest that the transcription of DR5 is controlled by Sp1 in 9–13 cells and that the HCV sub-genomic replicon enhances the transcription of DR5 via Sp1. We then co-transfected a plasmid expressing individual HCV nonstructural protein and a plasmid containing either DR4/−632 or DR5/−560 into Huh7 cells. Expression of the NS3/4, NS4B or NS5A protein increased DR5 promoter activity, and expression of the NS3/4A or NS4B protein increased DR4 promoter activity. However, the luciferase activity was too low, suggesting that the expression of these individual nonstructural proteins barely activates the DR4 and DR5 promoters (Fig. 5G).

### HCV-mediated up-regulation of DR4 and DR5 requires MEK1 activity

Sp1 is a member of a large family of transcription factors characterized by their affinity for GC-rich motifs. These transcription factors control the basal expression of housekeeping genes and genes lacking or containing a TATA box [28]. Sp1 activity is mainly regulated by post-translational modifications, with phosphorylation the most studied post-translational modification. Recent studies have shown that MAPKs, such as ERK and JNK, can phosphorylate Sp1 [29], [30], [31].

**Figure 6:**
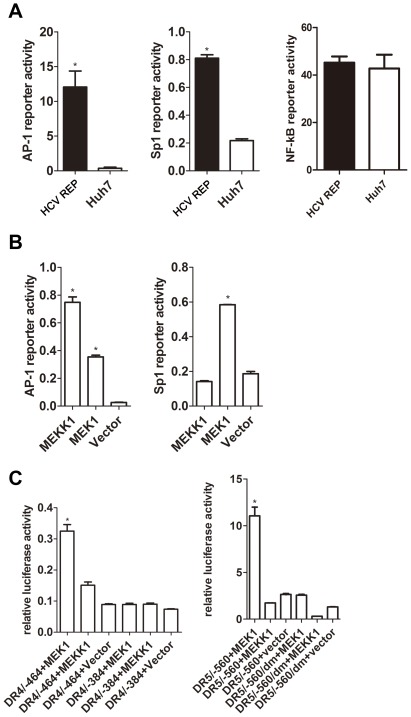
Over expression of MEK1 activates the DR4 and DR5 promoters. (A) The AP-1, Sp1 or NF-κB luciferase reporter plasmid (100 ng) was co-transfected with the *Renilla* luciferase reporter plasmid (100 ng) into 9–13 or Huh7 cells cultured in 24-well plates. 2 days post-transfection, luciferase activity was measured. (B and C) pMEK1-pRK or pMEKK1-pRK (600 ng) and the *Renilla* luciferase reporter plasmid (100 ng) were co-transfected with the (B) AP-1 or Sp1 reporter plasmid (100 ng) or (C) the indicated DR4 or DR5 reporter plasmid (100 ng) into Huh7 cells cultured in 24-well plates. 2 days post-transfection, luciferase activity was measured. The data are presented with the SD from three independent experiments, and statistical significance was calculated by *t* test, * indicates a *p* value less than 0.05.

Macdonald and colleagues (2003, 2005) have reported that the HCV NS5A protein can inhibit AP-1 activity by perturbing Ras-ERK signaling [32], [33]. Because the DR5 promoter contains two Sp1 binding sites, we studied the role of MAPKs in the HCV replicon-induced up-regulation of DR5. When compared with Huh7 cells, the AP-1 and Sp1 activity was increased in 9–13 cells, while the NF-κB activity was not changed (Fig. 6A). Furthermore, the over expression of MEK1 in Huh7 cells enhanced the transcription activity of AP-1 and Sp1, and the over expression of MEKK1 in Huh7 cells increased the transcription activity of AP-1 (Fig. 6B). These results suggest that HCV replication may activate MAPKs.

MEK1 is a dual specificity kinase that phosphorylates and activates ERK-1 and ERK-2 [34], which can activate AP-1 [29]. Additionally, MEKK1 can activate JNK [35], which also activates AP-1. Therefore, we investigated the relationship between the MAPKs and the expression of the TRAIL receptors. We co-transfected pDR4/−464 or pDR4/−384 with plasmids expressing MEK1 or MEKK1 into Huh7 cells and found that over expression of MEK1 increased the activity of pDR4/−464, but did not affect the activity of pDR4/−384. Similarly, over expression of MEK1 increased the activity of the DR5 promoter pDR5/−560 but not the pDR5/−560dm. In contrast, MEKK1 only slightly increased the DR4 promoter activity and did not affect the DR5 promoter activity (Fig. 6C). These results suggest that MEK1/ERK, but not MEKK1/JNK, is involved in the regulation of DR4 and DR5 expression. Furthermore, the region between −464 and −384 in the DR4 promoter and the Sp1 binding sites in the DR5 promoter were required for MEK1-mediated regulation of DR4 and DR5 activity.

**Figure 7:**
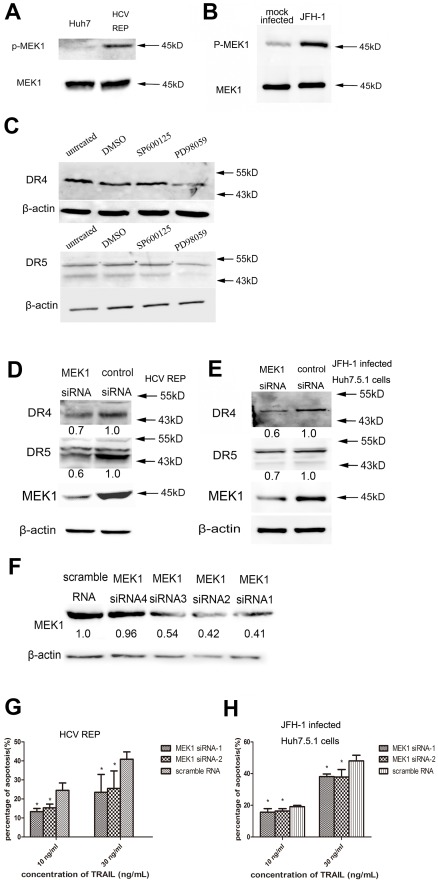
HCV-mediated increased DR4 and DR5 expression is MEK1 dependent. Huh7 and 9–13 cell lysates (A) or Huh7.5.1 and JFH-1 infected Huh7.5.1 cell (MOI 0.02) lysates (B) were subjected to western blot analyses using antibodies against phospho-MEK1 or MEK1. (C) Untreated 9–13 cells or 9–13 cells treated with 100 µM PD98059, 100 µM SP600125 or DMSO for 2 days were harvested and subjected to western blot analyses using antibodies against DR4 or DR5. (D) The MEK1-specific siRNA or control siRNA (100 pmol) was transfected into 9–13 cells cultured in 6-well plates. 2 days post-transfection, the expression of MEK1, DR4 and DR5 was determined using western blot analyses. (E) Huh7.5.1 cells were transfected with the MEK1-specific siRNA or control siRNA and infected with JFH-1 (MOI 0.02) 6 hr post transfection. 3 days post infection, the expression of MEK1, DR4 and DR5 was determined using western blot analyses. (F) Huh7.5.1 cells were transfected with indicated siRNA, and the expression of MEK1 was measured by using western blot 2 days later. (G) 9–13 cells were transfected with MEK1 siRNA1, MEK1 siRNA2 or scramble RNA, 3 days post transfection, cells were treated indicated concentration of TRAIL for 2 hr, and stained with annexin V and PI. The proportion of apoptotic cells was analyzed by using flow cytometry. (H) Huh7.5.1 cells were transfected with MEK1 siRNA1, MEK1 siRNA2 or scramble RNA, 6 hr later, cells were infected with JFH-1 (MOI 0.5), 3 days post infection, cells were treated indicated concentration of TRAIL for 2 hr, and stained with annexin V and PI. The proportion of apoptotic cells was analyzed by using flow cytometry. The data are presented with the SD from three independent experiments, and statistical significance was calculated by two-way ANOVA, * indicates a *p* value less than 0.05.

Activation of MEK1 occurs via phosphorylation of two serine residues at positions 217 and 221 [36]. Using an antibody against phosphorylated MEK1 (Ser217/221), we detected the phosphorylated form of MEK1 in 9–13 cells using western blot analyses. As shown in Fig. 7A, phosphorylation level of MEK1 was higher in 9–13 cells when compared with Huh7 cells, suggesting that HCV replication activates MEK1. Furthermore, we investigated whether inhibition of MEK1/ERK or MEKK1/JNK reversed the HCV-mediated up-regulation of DR4 and DR5. Treatment with PD98059, a MEK1-specific inhibitor, reversed the high expression of DR4 and DR5 in 9–13 cells, but a specific inhibitor of JNK, SP600125, had no effect (Fig. 7C). Knockdown of MEK1 using siRNA also decreased the expression of DR4 and DR5 (Fig. 7D). Moreover, JFH-1 infection stimulated MEK1 activity in Huh7.5.1 cells (Fig. 7B), and knockdown of MEK1 using siRNA prior to infection reversed the HCV-mediated up-regulation of DR4 and DR5 (Fig. 7E). Taken together, these data indicate that MEK1 has a critical role in the HCV-mediated up-regulation of DR4 and DR5. To confirm that knockdown of MEK1 could decrease the sensitivity to TRAIL-induced apoptosis, we tested other four pairs of siRNA of MEK1, and choose two of them to perform the next experiment (Fig. 7F). As shown in Fig. 7G and 7H, knockdown of MEK1 weakened the apoptosis induced by TRAIL both in 9–13 cells and JFH-1 infected Huh7.5.1 cells.

**Figure 8:**
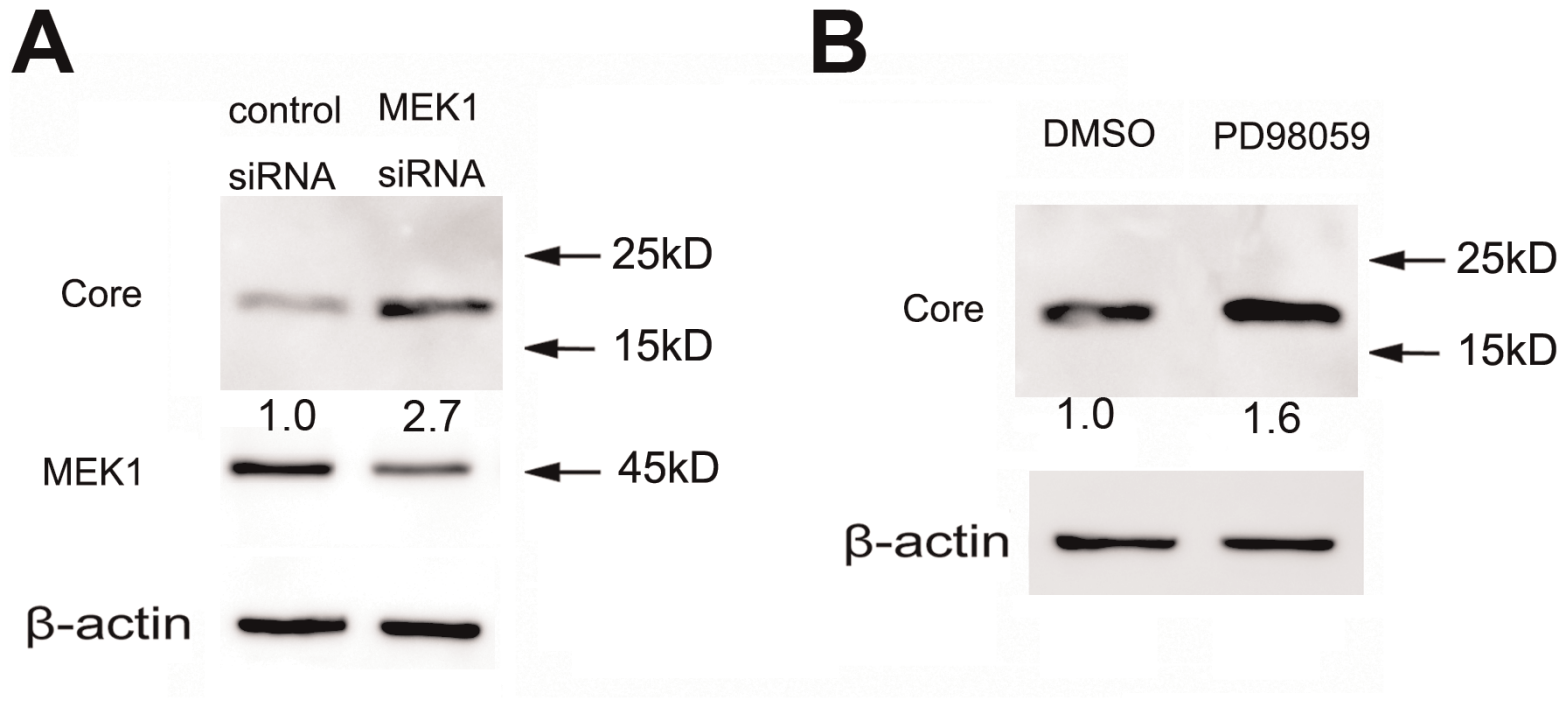
Inhibition of MEK1 increases HCV replication. Huh7.5.1 cells were transfected with a MEK1-specific siRNA (A) or treated with 10 µM PD98059 (B) and 6 hr post-transfection or treatment, infected with JFH-1 (MOI 0.02). 3 days post-infection, the expression of the HCV core protein was detected using western blot.

Inhibition of MEK1 activity by its specific inhibitor, PD98059, can enhance the replication of HCV [37], [38]. Therefore, we tested if inhibition of MEK1 enhanced HCV replication. As shown in Fig. 8, both PD98059 and a MEK1-specific siRNA increased JFH-1 replication in Huh7.5.1 cells. Together, these results suggest that MEK1 may be stimulated to inhibit HCV replication when cells are infected by HCV.

## Discussion

The relationship between HCV infection and TRAIL and its receptors has been previously studied. Clinical evidence has shown that HCV infection is related to the up-regulation of DR4 and DR5. Saitou et al. (2005) have reported that TRAIL, DR4 and DR5 are over expressed in the cytoplasm and on the surface of hepatocytes from patients chronically infected with HCV [18]. Volkmann and colleagues (2007) reported that TRAIL alone triggers massive apoptosis and caspase activation in tissue explants from patients with liver steatosis or HCV infection and that the enhanced sensitivity of diseased liver is associated with the increased expression of DR4 and DR5 [19]. Yano et al. (2003) reported that some HCV-related HCC cases have increased caspase 3 activity and expression of DR4 and DR5 in the tumor tissue [20]. However, Mundt et al. (2003) reported that DR4 and DR5 are not up-regulated in chronic hepatitis-associated cirrhosis [21]. It also remains controversial if TRAIL receptors are up-regulated by HCV infection in a cell culture system. Lan et al. (2008) reported that DR4 and DR5 are not up-regulated in JFH-1 infected Huh7.5 cells, whereas Zhu et al. (2007) reported that the expression of DR4 and DR5 is elevated in a JFH-1 infected hepatoma cell line, named by LH86 [4], [23]. Therefore, the mechanism underlying the HCV-mediated regulation of the TRAIL receptors remains unclear.

MEK1 is a dual specificity kinase that phosphorylates and activates ERK1/2 by phosphorylating threonine and tyrosine residues [34], and ERK1/2 can phosphorylate Sp1 [29]. Schmitz and colleagues (2008) reported that the increased phosphorylation of ERK1/2 (pERK) is correlated with HCV-induced cirrhosis and that approximately half of HCV-infected HCCs are pERK1/2 positive [39]. These findings are similar to the results in this study where phosphorylation of MEK1 was found to be increased in 9–13 cells and JFH-1 infected Huh7.5.1 cells (Figs. 7A and 7B), suggesting that MEK1/ERK are activated by HCV. Moreover, the current study demonstrates that the inhibition of MEK1 activity or expression reverses the HCV-mediated up-regulation of DR4 and DR5 (Figs. 7C, 7D and 7E). JNK can also phosphorylate Sp1 to increase the stability of Sp1 [40]. The present study showed that the over expression of MEKK1, which can strongly activate JNK, did not increase DR5 transcription but slightly increased DR4 transcription (Fig. 6C). Bild et al. (2002) reported that the over expression of MEKK1 can induce expression of DR4 and that MEKK1 can increase DR4 expression at the RNA level approximately 1.5 fold [41]. In the present study, MEK1 increased the DR4 promoter activity to a greater extent. Treating 9–13 cells with the JNK inhibitor, SP600125, did not abolish the expression of DR4 or DR5. These results indicate that the HCV-induced up-regulation of DR4 was mediated by MEK1 but not MEKK1.

The results of the present study showed that Sp1 activity was critical for HCV-induced up-regulation of DR5, but we were unable to identify the transcription factor that controls DR4 transcription in 9–13 cells. Moreover, point mutations at the two putative AP-1 binding sites in the DR4 promoter (−410/−404 bp or −552/−545 bp) did not influence the activity of the DR4 promoter (Fig. 5B). Previous studies have demonstrate that DR4 is regulated by both NF-κB and p53 [42], [43]. However, NF-κB activity was not significantly different in 9–13 and Huh7 cells (Fig. 6A). Importantly, the Huh7 cell line carries a single p53 mutation (A:T→G:C at codon 220) and is p53 defective [44], [45]. Thus, it is possible that other transcription factors are involved in the HCV-mediated up-regulation of DR4, and further research is needed to identify the transcription factors involved in HCV-regulated DR4 transcription.

Because both the sub genomic replicon and full-length virus increased the expression of DR4 and DR5, the structural proteins may not be involved in the regulation of the TRAIL pathway. We examined which HCV nonstructural proteins contributed to the up-regulation of DR4 and DR5. Although we found that the expression of NS3/4A or NS4B had a modest effect on the DR4 promoter activity and that the expression of NS3/4A, NS4B or NS5A had a similar modest up-regulation of the DR5 promoter activity, these effects were much weaker than those observed when the replicon was present (Fig. 5G). These results suggest that either the replication of the HCV genome or multiple HCV proteins may be needed to fully induce DR4 and DR5 expression.

Treating cells with the MEK1 inhibitor, PD98059, enhances HCV replication [37], [38]. Moreover, the Ras-ERK pathway has an important role in the IFN-γ and oxidative stress-induced anti-HCV effect, and activation of the Ras-ERK pathway either by EGF stimulation or over expression of Ras can suppress HCV replication [46], [47]. Therefore, the Ras-ERK pathway is a negative regulator of HCV replication. However, Gretton et al. (2009) reported that different MEK1/2 inhibitors have different effects on HCV replication: PD98059 causes a modest increase in HCV replication, but U0126 and PD184352 inhibit HCV replication. Moreover, they showed that neither EGF nor a high concentration of PD98059 affects HCV NS5A protein expression in cells transfected with the HCV replicon and that the transfection of a dominant negative MEK1 mutant inhibits HCV replication [48]. These results demonstrate that low MEK/ERK activity is required for HCV replication because PD98059 is less effective than U0126 in inhibiting MEK/ERK activity. In this study, we found elevated MEK1 activity in HCV-infected cells (Fig. 7B). Partial inhibition of MEK1 by PD98059 or a siRNA significantly increased the replication of JFH-1 (Fig. 8). Together, these results suggest that MEK1 with high activity is a negative regulator of HCV and is stimulated by HCV infection. This study demonstrates that HCV replication sensitizes host cells to TRAIL-induced apoptosis by up-regulating the DR4 and DR5 via a MEK1-dependent pathway. These findings may help to elucidate the mechanism underlying the HCV-mediated sensitization of cells to TRAIL and, thus, may help to further unravel the pathogenesis of HCV infection and provide new therapeutic targets for the treatment of HCV infection.

## Materials and Methods

### Plasmids and reagents

Reporter plasmids were constructed using the pGL3 vector containing a firefly luciferase open reading frame (Promega). The human DR4 and DR5 promoters were cloned from DNA extracted from Huh7 cells using the PUREGENE DNA Purification System, as recommend by the manufacturer (Gentra). PCR for the DR4 promoter was performed with following sense primers: DR4/−1156 (sense, 5′-GCAGATCTGCCCGGTCGAAAAGAGTCTTTTCAA-3′), DR4/−632 (sense, 5′-GCCTCGAGCCAAAACAGTGAAACCCCCGTCTC-3′), DR4/−541 (sense, 5′-GCCTCGAGGCTGAGGCAGGAAAATCGCTTGAAC-3′), DR4/464 (sense, 5′-GCCTCGAGGGGCGACAGAGCTTGACTCCATCTC-3′), DR4/−384 (sense, 5′-GCCTCGAGGGAGGCCGTAAAAGCCTCTTAGAGG-3′), and DR4/−349 (sense, 5′- GCCTCGAGCAGTGGCCTCTGTGTCCTTCATTCC-3′). The following antisense primer was used for all of the above reactions for DR4: 5′-GCAAGCTTCATCCTGCCAGGTCAATCCAAGAAG-3′. PCR for the DR5 promoter was performed with following sense primers: DR5/−1192 (sense, 5′-GCCTCGAGCACCAGAAGGAAGAAACTCCGAACA-3′), DR5/−560 (sense, 5′-GCCTCGAGAGAGAAGGAGAGAACAGAAGGGGCA-3′), DR5/−220 (sense, 5′-GCCTCGAGAGTTGCACATTGGATCTGATTCGCC), and DR5/−115 (sense, 5′-ACCTCGAGGGCCGGAGAACCCCGCAATCTCTGC). The following antisense primer was used for all of the above reactions for DR5: 5′-GCAAGCTTGGCGGTAGGGAACGCTCTTATAGTC-3′. Point mutations were introduced into pDR4/−632 and pDR5/−560 using PCR-based site-directed mutagenesis and the following primers: 5′-ATCCCAGTTACTTGGGAGGC-3′ and 5′-GCCTCCCAAGCAGCTGGGAT-3′ for the −552/−545 AP-1 binding site in DR4 promoter to generate pDR4/−632/−547m, 5′-GGCAGGCTGAATTGCTCGCC-3′ and 5′-GGCGAGCAATTCAGCCTGCC-3′ for the −410/−404 AP-1 binding site in DR4 promoter to generate pDR4/−632/−410m, 5′-ATTCGCCATGTACCGAATGA-3′ and 5′-TCATTCGGTACATGGCGAAT-3′ for the −195/−189 Sp1-binding site in the DR5 promoter to generate pDR5/−560/−195m and 5′-AGCCGCGATGATCCAAGTCA-3′ and 5′-TGACTTGGTACATCGCGGCT-3′ for the −149/−143 Sp1-binding site in the DR5 promoter to generate pDR5/−560/−149m. The pDR4/−632/dm plasmid was mutated both at the −552/−547 and −410/−404 sites in the DR4 promoter and the pDR5/−560/dm plasmid was mutated both at the −195/−189 and −149/−143 sites in the DR5 promoter (the ATG translation initiation site of DR4 and DR5 was designed as +1). The Sp1 reporter plasmid was constructed by introducing five Sp1-binding sites (5′-TGGGCGGGGC-3′) into the pGL3 vector. The AP-1 reporter plasmid (pAP-1-pGL3), NF-κB reporter plasmid (pNF-κB-pGL3), MEK1 expression plasmid (pMEK1-pRK) and MEKK1 expression plasmid (pMEKK1-pRK) were kindly provided by Dr. Hongbin Shu from Wuhan University. The MEK1 inhibitor, PD98059, was purchased from Cell Signaling Technology, and the JNK inhibitor, Sp600125, was purchased from Sigma.

### Cell lines, virus and DNA transfection

The human hepatoma cell lines, Huh7 and 9–13, were kindly provided by Dr. Ralf Bartenschlager from Universitätsklinikum Heidelberg. Both cell lines were cultured in DMEM supplemented with 10% fetal bovine serum (GIBCO) at 37°C, and the 9–13 cells were maintained in medium supplemented with 250 µg/mL G418 (Sigma). All transfection was performed using Lipofectamine 2000 (9–13 and Huh7 cells) (Invitrogen) or FuGENE HD (Huh7.5.1 cells) (Roche), as recommended by the manufacturer. JFH-1 and Huh7.5.1 cells were kindly provided by Prof. Hongbin Shu (Wuhan University, Wuhan, China). The virus titer was 10^4^ and 10^6^ ffu/mL, and Huh7.5.1 cells were infected at an MOI of 0.02 or 0.5.

### Establishment of HCV-cured cells

HCV-cured cells were established according to the method described by Blight et al. [49]. Briefly, 9–13 cells were treated with 100 IU/mL IFN-α for four passages. Clearance of the HCV replicon was confirmed by the loss of G418 resistance and NS3/4A expression.

### Caspase 3 activity assay

A Caspase 3 Activity Assay Kit (Beyotime) was used to measure caspase 3 activity. Huh7 and 9–13 cells were treated with 50 ng/mL TRAIL for 2 hr, and the cells were then lysed. The caspase 3 substrate (Ac-DEVD-*p*NA) was then added to the samples, after incubation at 37°C for 2 hrcaspase 3 activity was measured by detecting the absorbance at 405 nm.

### Luciferase activity assay

Cells cultured in 24-well plates were co-transfected with 50 or 100 ng of the *Renilla* luciferase reporter plasmid and 100 ng of the indicated *Firefly* luciferase reporter plasmids. 2 days post-transfection, cells were harvested and lysed in the a buffer containing 20 mM Tris-HCl (pH 7.4), 150 mM NaCl, 1 mM EDTA, 1% Triton X-100 and a protease inhibitor cocktail (Calbiochem). *Firefly* and *Renilla* luciferase activity was detected using a Dual Luciferase Reporter Assay System (Promega), as recommended by the manufacturer. *Firefly* luciferase activity was normalized using *Renilla* luciferase activity.

### Real-time PCR

Total cellular RNA was purified from cultured cells using TRIZOL (Invitrogen), as recommended by the manufacturer. Reverse transcription was performed using random primer and Moloney murine leukemia virus reverse transcriptase (Promega), and the cDNA was used as a template for real-time PCR. Real-time PCR was performed using a SYBR master mix (TOYOBO) and an ABI7300. The PCR conditions were as follows: 95°C for 60 s followed by 40 cycles of 95°C for 15 s, 60°C for 15 s, and 72°C for 45 s. The expression of DR4, DR5, Sp1 and GAPDH (as an internal control) was measured in 9–13 and Huh7 cells using the following primers: DR4 (sense, 5′-CTCGCAGTCCGCTTTCGTGT-3′, and antisense, 5′-CAGCATCAGAGTCGCAGTGG-3′), DR5 (sense, 5′-AAGACCCTTGTGCTCGTTGT-3′, and antisense, 5′-AGGTGGACACAATCCCTCTG-3′), DcR1 (sense, 5′-GGTGTGGATTACACCAACGCTTC-3′, and antisense, 5′-CTGACACACTGTGTCTCTGGTC-3′), DcR2 (sense, 5′-CTGCTGGTTCCAGTGAATGACG-3′, and antisense, 5′-TTTTCGGAGCCCACCAGTTGGT), Sp1 (sense, 5′-ATTGAGTCACCCAATGAGAACAG-3′, and antisense, 5′-CAGCCACAACATACTGCCC-3′), and GAPDH (sense, 5′-CACTCAGCCGCATCTTCTTT-3′, and antisense, 5′-ACGACCTAATCCGTTCACTC-3′).

The DR4, DR5, DcR1, DcR2 and Sp1 levels in each sample were normalized based on the GAPDH level in each sample.

### RNA interference

The Sp1-specific siRNA was purchased from Santa Cruz (sc-29487). The MEK1-specific siRNA was purchased from Genepharma (sense, 5′-GAGGUUCUCUGGAUCAAGUTT-3′, and antisense, 5′-ACUUGAUCCAGAGAACCUCTT-3′) and QIAGEN MEK1 siRNA1 (TTGTGAATAAATGCTTAATAA), siRNA2 (CTGGAAGAATTCCTGAACAAA). The siRNA (100 pmol/well) was transfected into cells cultured in 6-well plates using Lipofectamine 2000 (Invitrogen) or 37.5 ng/well into cells cultured in 24-well plates using Hiperfect transfection reagent (QIAGEN). 2 days post-transfection, the RNA and protein levels were measured using real-time PCR and western blot analyses, respectively.

### Induction of apoptosis by TRAIL

Cells were seeded (60–70% confluent) in 12-well plates. Twenty-four hr after plating or 3 days post transfection and infection the cells were treated with recombinant human TRAIL (R&D Systems) for 2 hr. Alternatively, 9–13 cells were incubated in the presence or absence of 20 µM Z-VAD-FMK (a pan-caspase inhibitor; R&D Systems) for 1 hr prior to treatment with TRAIL. Subsequently, the cells were harvested, washed with PBS and stained with annexin V (Bender MedSystems), as recommended by the manufacturer. The proportion of apoptotic cells was determined using flow cytometry.

### Western blot analysis

Cells were lysed in a buffer described above in “Luciferase activity assay” section. After two freeze/thaw cycles, cell debris was removed by centrifugation. Equal amounts of the protein samples were resolved by 12% SDS-PAGE, and the proteins were transferred onto a nitrocellulose membrane (Amersham Biosciences). The membrane was probed using a polyclonal antibody against HCV NS3/4A protein (produced by our laboratory), core protein (Santa Cruz sc-57800), human DR4 (Abcam ab8414), DR5 (Sigma D3938), Sp1 (Santa Cruz sc-59), MEK1 (Cell Signaling Technology #2352), phospho-MEK1/2 (Cell Signaling Technology #9121) or a mouse monoclonal antibody against β-actin (Santa Cruz). Densitometric analyses were performed in some results by showing the ratio to β-actin.
